# Nurses on the frontline of health care in the escalating context of climate change: Climate‐related extreme weather events, injustice, mental health and eco‐anxiety

**DOI:** 10.1111/jan.15838

**Published:** 2023-08-28

**Authors:** Kim Usher AM, Kylie Rice, Syadani Riyad Fatema, Kisani Louise Upward, Rikki Jones

**Affiliations:** ^1^ School of Health University of New England Armidale New South Wales Australia; ^2^ School of Psychology University of New England Armidale New South Wales Australia

## 1 EXTREME WEATHER EVENTS

Natural disasters including extreme weather events are a stark reminder of what will most likely become our future norm due to the impact of global warming and climate change. Due to climate change, more intense and frequent extreme weather events are happening globally (Longman et al., 2023). Recently in Australia, we have experienced extreme weather events such as droughts, floods, bushfires, storms, cyclones and tornadoes, at an unprecedented rate (Ghosh et al., 2022), and similar experiences have occurred globally (Fatema et al., 2021). The outcomes of these extreme weather events have had significant consequences for human health and well‐being, with the degree of emotional distress and anxiety experienced related to how these events have altered or threatened an individual's environment and way of life (Ingle & Mikulewicz, 2020). Extreme weather event experiences are associated with recognized mental and physical health impacts (e.g., Fatema et al., 2023), and they are likely to provoke eco‐anxiety. Eco‐anxiety occurs when people experience negative emotional responses such as distress and a sense of despair related to climate change (Coffey et al., 2021). Climate change disproportionately affects the most vulnerable and marginalized people in society (Hayes et al., 2018). People from more disadvantaged regions have reported experiencing higher levels of eco‐anxiety, or anxiety related to climate change, whether they have had direct experience of extreme weather events or not (Patrick et al., 2022). These findings collectively highlight the importance of the consideration of eco‐anxiety by practitioners and researchers within mental health assessments, over and above traditionally recognized mental health disorders. This may be particularly important for people from disadvantaged regions, vulnerable groups or those who have experienced extreme weather events. This paper addresses these issues and provides an overview of the related evidence. Finally, it makes recommendations for health service change.

## 2 MENTAL HEALTH AND ECO‐ANXIETY

Climate variability can have disastrous effects on human societies causing an increasing burden on health and mental health, contributing to an already strained health care system. Climate change increases the magnitude of extreme weather‐related events and the increased frequency of events offers little time for recovery (Crandon et al., 2022). It has been argued that natural disasters and extreme weather events (such as ongoing droughts, unprecedented rainfall and severe storms, acid rain, large‐scale fires, glacier melting, heatwaves and ocean acidification) has an increased impact on mental health, beyond the psychosocial impact of the normal seasonal weather variations (Patrick et al., 2022). Mental health effects of extreme weather events include mood disturbances such as depression, irritability, anxiety, substance use, post‐traumatic stress disorder (PTSD), increased aggression and increased suicide rates (Fatema et al., 2021). As a result of the increasing awareness of climate‐related mental health, the term eco‐anxiety has been adopted to describe the anxiety associated with the changing climate, increased by the lived experience of extreme weather events. Eco‐anxiety is characterized by ‘…severe and debilitating worry about climate and environmental risks…’ (Ingle & Mikulewicz, 2020, p. e128) and can result in negative emotional responses such as distress and a sense of despair (Coffey et al., 2021). It is important to distinguish eco‐anxiety from other emerging psychoterratic terminologies, such as eco‐grief, which refers to bereavement and yearning related to losses from the destruction of the natural environment, such as ecosystems, landscapes, livelihood and way of life (Comtesse et al., 2021). As stated by Patrick et al. (2022), ‘eco‐anxiety, in contrast to other eco emotions such as eco‐grief and eco‐despair, highlights the mental health impacts of future‐orientated worry about the potential impacts of climate change’ (p. 7). As such, eco‐anxiety is a future‐focused reaction to anticipated threats of climate change, that includes an emotional response (e.g., fear, distress), cognitive components (e.g., worry) and physiological arousal (Comtesse et al., 2021). Lower levels of eco‐anxiety may be a fundamentally adaptive response to ecological loss or future‐focused impending threats, resulting in the facilitation of positive behavioural change. Higher levels of eco‐anxiety can be maladaptive and debilitating and can be associated with a broad spectrum of health concerns (Boluda‐Verdú et al., 2022). Similarly, Ingle and Mikulewicz (2020) affirm that emotional distress and mental health issues related to climate change are less visible than physical impacts. Hence the importance of ensuring that eco‐anxiety and other climate change‐related mental health issues are recognized.

## 3 VULNERABLE POPULATIONS AND JUSTICE

Studies on the impacts of disasters and extreme weather events have revealed that vulnerable populations are at a disproportionately greater risk compared with other groups (Benevolenza & DeRigne, 2019). Particularly, these people are at an increased risk of displacement (homelessness), increased negative health outcomes (both physical and mental health) and decreased economic stability (Fatema et al., 2021). Some of the more vulnerable groups at risk include people with lower socio‐economic status, children, older people and some minority groups (Benevolenza & DeRigne, 2019; Fatema et al., 2021), and those who depend on the land for survival are also susceptible (Patrick et al., 2022). Women are considerably more vulnerable to the impact of disasters (Fatema et al., 2023) and a recent systematic review revealed that many women are prone to physical and emotional issues after disasters, especially those from lower socio‐economic locations, especially in rural and remote areas (Fatema et al., 2021). Farmers in rural and remote areas are also especially vulnerable and disadvantaged by the impacts of extreme weather‐related events (Comtesse et al., 2021); these events can result in serious mental health issues and adverse effects for farmers, as their livelihoods depend on the ecosystem (Patrick et al., 2022). Indigenous Peoples are also at increased risk from extreme weather events. For Indigenous Peoples, the emotional experience may be more intense due to their connection to Country and exacerbated by the environmental damaged caused by the changing climate. These experiences can impact Cultural practices, Cultural connections and social, emotional and physical well‐being, as the land for many Indigenous Peoples is necessary for survival (Standen et al., 2022).

It is essential to recognize the differential impacts of climate change for these vulnerable groups and communities, in terms of both impacts and ability to respond. Within the response to extreme weather events that is developing around the world, climate justice highlights the inequities in climate change; identifying how higher income countries historically have increased responsibility yet may face the least adverse events from climate change (Ingle & Mikulewicz, 2020). Furthermore, many of these vulnerable communities and groups may have less potential to effectively respond or adapt to the impacts of extreme weather events, due to factors such as socio‐economic disadvantage, health sensitivities and political determinants (Standen et al., 2022). Therefore, climate justice highlights that vulnerability, impact, responsibility and ability to respond are all differentially distributed between individuals and communities (Ingle & Mikulewicz, 2020). Recognition of these inherent differences needs to guide response to individual and community needs.

## 4 CLINICAL IMPLICATIONS

Practitioners and mental health systems need to be oriented towards the identification, assessment and treatment of climate‐related mental health symptoms (Patrick et al., 2022). In addition to extreme weather event experiences being associated with recognized mental and physical health impacts (e.g., Fatema et al., 2023), they are likely to also be eco‐anxiety provoking. Specifically identifying eco‐anxiety that develops post‐disaster and persists long term, may be an essential clinical feature for assessment and treatment, in addition to currently recognized post‐disaster negative effects on mental health. Furthermore, people from more disadvantaged regions have been found to experience higher levels of eco‐anxiety, whether they have had direct experience of extreme weather events or not (Patrick et al., 2022). Thus, ‘people aware of current and future threats from climate change may feel fear and anxiety about potential negative outcomes for their future and the planet’ (Boluda‐Verdú et al., 2022, p. 2). These findings collectively highlight the importance of the consideration of eco‐anxiety by practitioners and researchers within mental health assessments, over and above traditionally recognized mental health disorders. This may be particularly important for people from disadvantaged regions, vulnerable groups or those who have experienced extreme weather events. Given that eco‐anxiety may also be a manifestation of underlying mental health issues and has been associated with other health concerns (Boluda‐Verdú et al., 2022), eco‐anxiety could potentially present as a primary or secondary symptom, which may serve to precipitate, maintain or exacerbate other mental and physical health symptoms. Thus, accurate case conceptualisation is essential to understand the idiographic presentation of eco‐anxiety and its interaction with other mental health concerns in clinical practice. Attention should be given to clinically relevant eco‐anxiety symptoms such as maladaptive worry, cognitive impairment and/or functional impairment in one or more domains (Patrick et al., 2022). To facilitate the integration of eco‐anxiety within mental health assessment and screening, valid measurement tools with normative data on eco‐anxiety levels and clinical cut‐off scores are needed, for application in both practice and research. It is essential that these tools are developed with and validated for use in disadvantaged regions and with vulnerable subpopulations.

In view of climate injustice, particular attention needs to be on equity of mental health services for disadvantaged regions, vulnerable individuals and communities (Ingle & Mikulewicz, 2020). Given the shortage of mental health services in rural and remote areas (Fatema et al., 2021), and the grave consequences of climate change for rural community members and their livelihoods, innovative approaches are required to mitigate adverse outcomes and to ensure that all people impacted by these events can access required services as needed. In addition to improving equity and access to mental health services for those most disadvantaged, service provision should encompass attention to the recognition and alleviation of eco‐anxiety and climate change‐related mental health issues.

## 5 FINAL TAKEAWAY

Mental health professionals, including nurses, have an important role to play in managing the impact of climate change on mental health. Nurses are in the frontline of healthcare services and will likely carry a large share of the increased mental health burden of climate change. On a clinical level, nurses have a crucial role in the identification and treatment of mental health impacts of climate change and as first responders to increased presentations from extreme weather events. On a community level, healthcare services need to prepare for climate change‐related increased demand through training, workforce capacity, education, and community‐level intervention (Crandon et al., 2022). Nurses are crucial in all of these aspects and will be increasingly called upon to respond to these service priorities, and to the increased community need. On a systemic level, all health practitioners need to facilitate and advocate for sustainable practices, service readiness and climate action (Crandon et al., 2022). Nurses are on the frontline of healthcare and are pivotal in addressing this escalating mental health demand, on individual, community and systemic levels.

## AUTHOR CONTRIBUTIONS

KU and KR conceived the idea and led the writing; SRF, KLU and RJ contributed to the idea and the writing of the manuscript.

## CONFLICT OF INTEREST STATEMENT

The authors have no conflicts of interest to declare.

## Data Availability

Not applicable.
